# Nanoemulsions and Solid Microparticles Containing Pentyl Cinnamate to Control *Aedes aegypti*

**DOI:** 10.3390/ijms241512141

**Published:** 2023-07-29

**Authors:** Addison R. Almeida, Waldenice A. Morais, Nicolas D. Oliveira, Wilken C. G. Silva, Ana P. B. Gomes, Laila S. Espindola, Marianna O. Araujo, Renata M. Araujo, Lorena C. Albernaz, Damião P. De Sousa, Cicero F. S. Aragão, Leandro S. Ferreira

**Affiliations:** 1 Laboratório de Farmacotécnica, Department of Pharmacy, Federal University of Rio Grande do Norte, Natal 59012-570, Brazil; addison.ribeiro@ufrn.br (A.R.A.); waldenice.lima@ufrn.br (W.A.M.); nicolas_dantas_oliveira@hotmail.com (N.D.O.); wilkencesar.wc@gmail.com (W.C.G.S.); 2 Laboratório de Controle de Qualidade de Medicamentos, Department of Pharmacy, Federal University of Rio Grande do Norte, Natal 59012-570, Brazil; ana.pbgomes@gmail.com (A.P.B.G.); cicero.aragao@yahoo.com.br (C.F.S.A.); 3 Laboratory of Phamacognosy, Brasília University, Campus Universitário Darcy Ribeiro, Brasília 70910-900, Brazil; darvenne@gmail.com (L.S.E.); lorena.albernaz@gmail.com (L.C.A.); 4 Laboratory of Pharmaceutical Chemistry, Federal University of Paraíba, João Pessoa 58050-085, Brazil; marianninhaoliveira@yahoo.com.br (M.O.A.); damiao_desousa@yahoo.com.br (D.P.D.S.); 5 Chemistry Institute, Federal University of Rio Grande do Norte, Natal 59072-970, Brazil; renat.onca@gmail.com

**Keywords:** *Aedes aegypti*, larvicide, pentyl cinnamate, nanoemulsion, solid microparticles

## Abstract

The *Aedes aegypti* mosquito is a vector of severe diseases with high morbidity and mortality rates. The most commonly used industrial larvicides have considerable toxicity for non-target organisms. This study aimed to develop and evaluate liquid and solid carrier systems to use pentyl cinnamate (PC), derived from natural sources, to control *Ae. aegypti* larvae. The liquid systems consisting of nanoemulsions with different lecithins systems were obtained and evaluated for stability over 30 days. Microparticles (MPs) were obtained by the spray drying of the nanoemulsions using maltodextrin as an adjuvant. Thermal, NMR and FTIR analysis indicated the presence of PC in microparticles. Indeed, the best nanoemulsion system was also the most stable and generated the highest MP yield. The PC larvicidal activity was increased in the PC nanoemulsion system. Therefore, it was possible to develop, characterize and obtain PC carrier systems active against *Ae. aegypti* larvae.

## 1. Introduction

The *Aedes aegypti* mosquito is a carrier that plays a significant role in widespread arboviral disease epidemics across the globe, especially in tropical and subtropical regions [1], with dengue being the most prominent among them [2]. Additionally, in 2016, Zika caused a major public health crisis in Brazil, leading to numerous cases of microcephaly [3]. The breeding sites of these mosquitoes typically consist of stagnant water areas, allowing them to thrive in urban environments. Disrupting the vector’s biological cycle is the most effective control measure for preventing these diseases. Larvicidal control methods involve the application of chemical compounds in water environments, preferably with no harmful effects on wildlife and plant life [4].

An obstacle that hinders the effective control of this mosquito is the emergence of populations resistant to certain chemical or biological insecticides. Since the 1980s, temephos, an organophosphate-based compound, has been the most widely used insecticide against dengue-transmitting larvae and yellow fever [5]. However, reports of *Ae. aegypti*’s resistance to this insecticide has surfaced in various regions [5,6,7]. Some pyrethroid compounds have been utilized as an alternative to organophosphates due to their high efficacy against adult mosquitoes [8]. Nonetheless, laboratory tests have demonstrated that pyrethroids are highly toxic to fish, bees and aquatic arthropods such as lobsters and shrimps [9]. Moreover, these commercial insecticides and their by-products have had a significant environmental impact over the years, as they infiltrate different types of soil and contaminate surface water sources [10,11].

Exploring new larvicidal compounds offers a promising solution to address the challenges posed by the resistance to existing larvicides. Natural products emerge as a potential alternative source, considering that numerous insecticides have been derived from natural compounds [12]. Although different natural products or secondary metabolites classes have been evaluated, many studies focus on particularly plant essential oils or their constituents [13,14].

**Figure 1:**
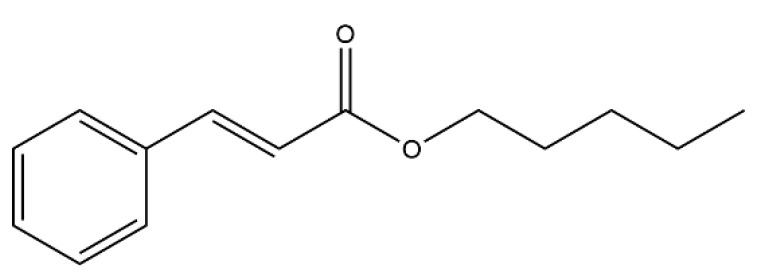
Pentyl cinnamate structure.

Cinnamic acid and its derivatives are natural bioactive compounds found in fruits and certain plant species, which are also known for their activity against *Ae. aegypti* and their remarkably cinnamic acid esters [15,16,17]. The pentyl cinnamate (PC), shown in Figure 1, which can be found in the essential oil of the aerial parts of *Piper pierrei* C.D.C. [18], has no reported toxic effects on the environment or humans. This compound is commonly used in cosmetic products and fragrances [19,20]. A study evaluating the activity of 17 cinnamic acid esters against the L4 larvae of *Ae. aegypti* highlighted pentyl cinnamate as one of the most active compounds [15]. However, pentyl cinnamate exhibits poor aqueous solubility, a common characteristic of new bioactive molecules and potential drugs [21]. To overcome this challenge, a strategy is to employ drug carrier systems such as nanoemulsions and nanoparticles [22].

Nanoemulsions are colloidal systems that protect, encapsulate and release the lipophilic substances derived from two immiscible liquids. They possess a higher solubilization capacity than simple micellar dispersions and exhibit superior kinetic stability compared to coarse emulsions. As a result, nanoemulsions have found applications in various industries, including cosmetics and pesticides, as they provide an aqueous base for organic products [23]. Numerous studies have demonstrated the effectiveness of utilizing nanoemulsions to enhance solubility and prolong the larvicidal activity of essential oils and volatile compounds such as limonene [24,25].

Solid carrier systems have several advantages, such as microbiological stability, a lower risk of chemical and biological degradation, not to mention lower logistics costs in terms of storage and transportation. Moreover, they are helpful systems when the controlled release of the bioactive is desired, as the mobility of a substance in a solid is considerably less than in a liquid oil [26]. The most common process to obtain solid microparticles is through spray drying. This method is popular because the functional characteristics and quality of the final product are not usually compromised during the production process [27]. A growing number of studies involve the evaluation of solid lipid nanoparticles, polymeric nanocapsules and solid lipid microcapsules for insecticide and repellent applications. The results demonstrate the viability of these approaches in terms of the biocompatibility of lipid components and increased and controlled release [28,29].

No previous reports regarding using pentyl cinnamate (PC) in nanoemulsions for larvicidal application against *Ae. aegypti* were found. Hence, this study presents an innovative aspect as it aims to obtain PC, produce the lipid nanoemulsions containing it, characterize them as well as evaluate their stability and activity against *Ae. aegypti*. Furthermore, solid microparticles were developed from these nanoemulsions, and their yields and compatibility were assessed.

## 2. Results and Discussion

### 2.1. Esterification Reaction and Product Analysis

Pentyl cinnamate (PC) was synthesized through an esterification reaction of cinnamic acid, yielding approximately 70%. The compound’s identity was confirmed through characterization using LC-MS/MS and ^1^H-NMR techniques. The high-resolution mass spectrum (Figure S1, Supplementary Materials) displayed an [M+H]^+^ ion consistent with PC. The ^1^H-NMR spectrum (Figure S2, Supplementary Materials) exhibited three signals between 7.28 and 7.53 ppm, which are indicative of hydrogen atoms attached to aromatic carbons. The integration of these signals, totaling five, indicated the presence of a monosubstituted aromatic ring. Two doublets with chemical shifts of 7.68 and 6.44 ppm, each integrating for 1H, and a coupling constant (*J*) of 16 Hz, were assigned to the hydrogens in the trans isomeric olefins. The signal at δ 4.20 ppm was attributed to the two methylene hydrogens directly attached to the ester group’s oxygen atom. Signals at δ 1.78, 1.72 and 1.38 ppm were assigned to the other methylene hydrogens in the alkyl chain, while the signal at δ 0.93 ppm, located in a region of high electromagnetic shielding, was attributed to the methyl hydrogens. Complete ^1^H NMR (300 MHz) and ^13^C NMR (75 MHz) data can be found in Table S2 (Supplementary Materials). Consequently, the ^1^H NMR and mass spectrometry results support the identification of the substance as pentyl cinnamate [15,30]. The infrared (IR) spectrum (Figure S3, Supplementary Materials) also exhibited the characteristics consistent with pentyl cinnamate. All spectroscopic and spectrometric spectra used for characterization are available in the supplementary material.

PC: yellow oil; yield 68.9% (126.9 mg, 0.58 mmol); TLC (hexane); Rf = 0.22; IR υmax (KBr, cm^−1^): 3065, 3032, 2959, 1714, 1639, 1579, 1450, 1311, 1171, 767; ^1^H NMR (CDCl_3_, 300 MHz): δH 7.70 (1H; d; *J* = 16.0 Hz), 7.55–7.53 (2H; m), 7.42–7.39 (3H; m), 6.44 (1H; d; *J* = 16.0 Hz), 4.29–4.20 (2H; m; *J* = 6.8 Hz), 1.83–1.68 (2H; quint; *J* = 6.8 Hz), 1.43–1.38 (4H; m), 0.93 (3H; t; *J* = 7.2 Hz); ^13^C-NMR (CDCl_3_, 75 MHz,): δC 14.1, 22.4, 28.2, 28.5, 64.8, 118.4, 128.1, 129.0, 130.3, 134.5, 144.6, 167.2. LC-MS (ESI-QTOF, positive ion mode) *m*/*z* 219.1394 [M+H]^+^ (calcd. for C_14_H_19_O_2_ *m*/*z* 219.1385, error 4.1 ppm).

### 2.2. Nanoemulsions Preparation and Analysis

A total of 36 nanoemulsions were produced through high-speed homogenization using an Ultra-Turrax instrument. NE1 was prepared using soy lecithin containing 20% phosphatidylcholine, NE2 with soy lecithin containing 50% phosphatidylcholine and NE3 with soy lecithin containing 70% phosphatidylcholine. No significant differences were observed among the nanoemulsions regarding phase separation or instability phenomena such as creaming or flocculation. The nanoemulsions exhibited small droplet sizes, with NE1 measuring 399 ± 66 nm, NE2 measuring 158 ± 3 nm and NE3 measuring 460 ± 76 nm. Dynamic light scattering (DLS) traces can be found in the supplementary material (Figure S4). These findings are consistent with previous studies, indicating that high-energy techniques, such as high-speed homogenization, result in nanoemulsions with improved versatility and high performance compared to low-energy techniques. This is attributed to the application of disruptive forces, including shear, turbulence and cavitation, which emulsify the phases into small droplets [31]. Previous research has successfully employed high-speed homogenization using instruments such as the Ultra-Turrax to obtain stable nanoemulsions for various applications, including lecithins alone or in combination with surfactants such as Tween 80 [32,33].

Three temperatures were tested during the emulsification process: 25, 50 and 60 °C, to evaluate their effect on the average droplet size and size distribution. Only NE1 exhibited a reduced droplet size with low PDI values at 60 °C. Regarding the proportion of soy lecithin used, the best results were obtained with the highest concentrations tested. NE2 achieved optimal results with a 5.0% surfactant proportion, NE3 with a 1.5% proportion and NE1 with a 3.0% surfactant proportion (1.5% lecithin and 1.5% Tween 80). Determining the appropriate ratio of lecithin and co-surfactant was crucial for achieving the desired droplet size, homogeneity and stability [23].

While ^1^H NMR analysis is not commonly used for the characterization of nanoemulsions and other dispersed systems, it is a sensitive technique that can provide valuable insights into the compatibility between the drug and the delivery system, as well as enable quantification, as demonstrated in a study on the encapsulation efficiency of ibuprofen in solid lipid nanoparticles (SLNs) using ^1^H NMR data [34]. ^1^H NMR analysis can also reveal supramolecular arrangements by evaluating parameters such as changes in chemical shift and line width [35]. In this study, no incompatibilities were observed between PC and the other components of the nanoemulsions, as indicated by the absence of new ^1^H signals suggesting hydrolysis or significant shifts in the signals of nanoemulsions containing PC, which indicates the efficient incorporation of PC in the nanoemulsions. The ^1^H spectra and tables containing the chemical shift data of the nanoemulsions are provided in the supplementary material (Figures S5–S7 and Table S3).

### 2.3. Larvicidal Activity of Pentyl Cinnamate and Nanoemulsions

PC and nanoemulsions showed larvicidal activity against *Ae. aegypti* L3 larvae. At 24 h, the mortality rate was 100% PC at 50 µg/mL, with an LC_50_ value of 19.9 µg/mL. Previous studies evaluated the larvicidal activity of Ae. aegypti from cinnamic acid esters such as methyl cinnamate (LC_50_ 35.4 µg/mL) in L3 larvae [17] and pentyl cinnamate (LC_50_ 36.0 µg/mL) in L4 larvae, PC being the compound that presented the best activity among the 17 esters [15]. When compared to the larvicidal activity results of the oils, extracts and isolated compounds obtained in previous work [15,36,37,38], the potential use of PC is favorable as a larvicidal agent due to its lower LC_50_ value.

In the residuality tests, PC was effective for up to 48 h at 50 µg/mL. Another characteristic observed in the larvae treated with PC, also observed in the larvae treated with methyl cinnamate [17], was their darkening. This could be the result of a lesion in the epithelial cells, changing the peritrophic matrix and absence of underlying epithelium, resulting in the release of hemolymph. This mechanism has been reported as the first defense in the larva’s attempt to eliminate toxic substances [39,40].

**Figure 2:**
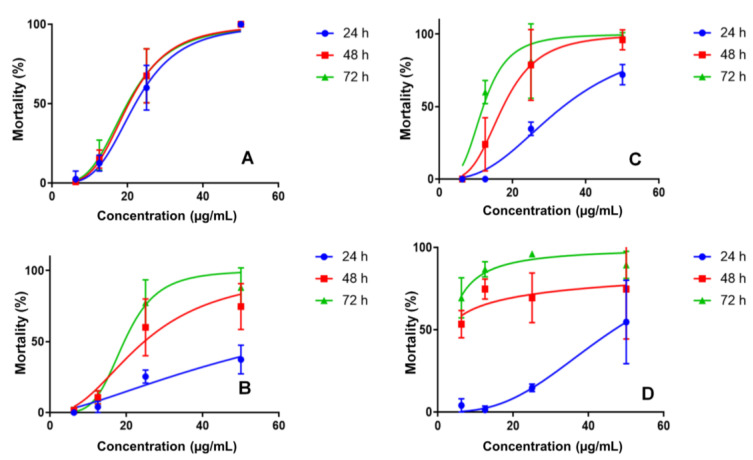
Larvicidal activity for PC (**A**), nanoemulsion NE1 (**B**), nanoemulsion NE3 (**C**) and nanoemulsion NE2 (**D**) in concentrations of 6.25–50 μg/mL.

The nanoemulsions developed to improve the bioavailability of the active compound showed different profiles regarding the mosquito larvae mortality. NE1 was evaluated at the same concentrations. At 50 µg/mL, a mortality rate of 37.3, 74.7 and 88% was observed after 24, 48 and 72 h, respectively. NE3 at 50 µg/mL had a mortality rate of 72.0, 96.0 and 98.7% after 24, 48 and 72 h, respectively, as shown in Figure 2.

The final nanoemulsion evaluated was NE2 (50 µg/mL), demonstrating a mortality rate of 54.7, 74.7 and 89.3% after 24, 48 and 72 h, respectively (Table S4, supplementary material). The larval mortality profiles indicated controlled PC release in nanoemulsions, which would be ideal for prolonging its activity in the environment. Nanoemulsions without PC caused *Ae. aegypti* L3 larvae mortality; however, at a much lower level than PC nanoemulsions, justifying its use. The observed mortality rates were 16.7%, 63.3% and 10.0% for NE1, NE2 and NE3, respectively, at a concentration of 25 µg/mL.

### 2.4. Nanoemulsion Stability

In the stability test, NE2 samples exhibited smaller droplet sizes and a lower PDI than those produced with other lecithins. Moreover, as indicated in Table 1, NE2 showed the least variation in droplet size, PDI and standard deviation among replicates, considering both time and temperature factors. Samples stored at 4 °C displayed less variation in droplet sizes, as higher temperatures tend to increase droplet movement, leading to greater collisions and triggering instability phenomena such as flocculation and coalescence [41]. Visual observations confirmed creaming in the NE1 samples, which correlated with the high variation in size and standard deviations. A foam layer was also observed in the NE3 samples, impeding the homogenization and higher standard deviation values.

Upon analyzing the data presented in Table 2, it becomes evident that the zeta potential values of the nanoemulsions remained relatively stable over time, consistent with findings from a previous study employing a similar methodology [42]. Negative zeta potential values are characteristic of nanoemulsions containing lecithins [43]. Among the nanoemulsions investigated, NE2 exhibited the highest stability throughout the 30 days. NE2 is a Phosal-based nanoemulsion formulated with PHOSAL 50 SA+, comprising 50% phosphatidylcholine, lysophosphatidylcholine and safflower oil. The compatibility be-tween safflower oil and lecithin in NE2 contributed to smaller droplet sizes and enhanced stability. Phosal is highly regarded as a solubilizer for water-insoluble compounds and for producing dispersed systems [44].

### 2.5. Spray Drying

The nanoemulsions prepared with different soy lecithins were subjected to spray drying using maltodextrin and L-leucine as adjuvants. The spray-dried powders obtained using maltodextrin exhibited higher yields (7.44%, 32.78% and 21.95% for MP1, MP2 and MP3, respectively) compared to those obtained with *L*-leucine (3.01%, 1.30% and 8.86% for MP1, MP2 and MP3, respectively). This can be attributed to the higher solubility of maltodextrin in water. Among the three lecithins tested, the samples formulated with soy lecithin containing 50% phosphatidylcholine showed the highest yields, exceeding 30%. A previous study utilizing maltodextrin as a drying agent achieved yields above 40% [45]. The resulting solid microparticles were characterized using FTIR, SEM and thermal analysis techniques such as DSC and TG. The decision to dry the nanoemulsions into solid microparticles was based on easier storage and transport as well the conservation improvement by the water removal.

### 2.6. Fourier Transform Infrared Spectroscopy—FTIR

**Figure 3:**
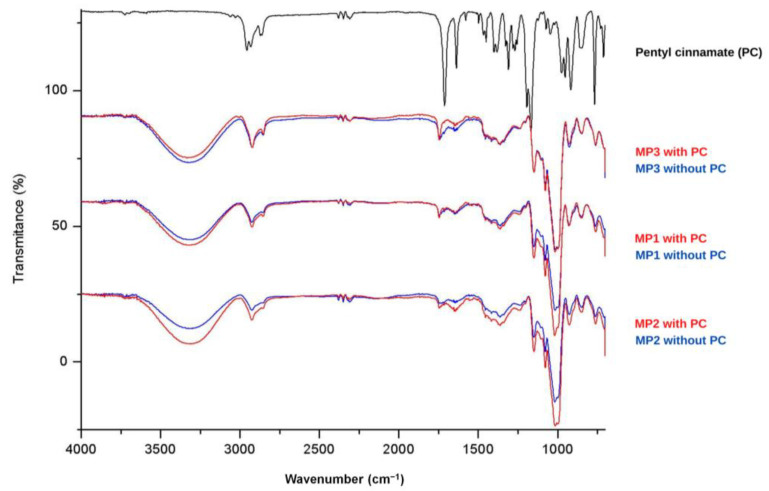
FTIR spectrum of pentyl cinnamate (PC) and MP1, MP2 and MP3 microparticles.

The solid microparticles MP1, MP2 and MP3, as well as placebo samples and PC, underwent Fourier transform infrared (FTIR) analysis, and the corresponding spectra are presented in Figure 3. The FTIR spectrum of PC (shown in black, Figure 3) exhibits characteristic peaks corresponding to the functional groups present in this molecule. In the region between 2850 and 2960 cm^−^^1^, characteristic bands related to the C-H bonds of CH, CH_2_ and CH_3_ groups are observed. Additionally, a prominent signal between 1740–1750 cm^−^^1^ indicates the stretching of the C=O bond in esters, with a slight shift to lower wave-numbers due to the resonance effects resulting from conjugation with the double bond and aromatic ring. At 1300 cm^−^^1^, a peak associated with the stretching of the C-O bond in esters is present, while in the range of 710–760 cm^−^^1^, two peaks typically associated with angular deformation of adjacent 5H in monosubstituted aromatic rings are observed. The appearance of new peaks or changes in the intensity of existing peaks can indicate interactions between the active ingredient and the components of the carrier systems [46].

Analysis of the spectra of the MP1, MP2 and MP3 placebo samples (shown in blue) reveals consistent signals, indicating similar composition among these systems. The broad band observed in the region of 3300 cm^−^^1^ can be attributed to the stretching of the O-H bond present in maltodextrin, which was used as a drying aid. The presence of peaks between 2850 and 2960 cm^−^^1^ suggests the presence of aliphatic C-H bonds. An intense signal at 1000 cm^−^^1^ may indicate the presence of the C-N bond in phosphatidylcholine, which is present in soy lecithins. In the spectra of the solid microparticles containing PC (shown in red), a slight increase in the intensity of the signals near 1716 cm^−^^1^ and 1300 cm^−^^1^ is observed, corresponding to the C=O and C-O bonds of the incorporated ester. Therefore, the analysis of FTIR spectra was valuable in characterizing the signals related to PC and the microparticles MP1, MP2 and MP3. However, it was impossible to observe significant changes in the characteristic signals of each component, indicating limited interactions between the constituents of the systems and the ester.

### 2.7. Scanning Electron Microscopy

**Figure 4:**
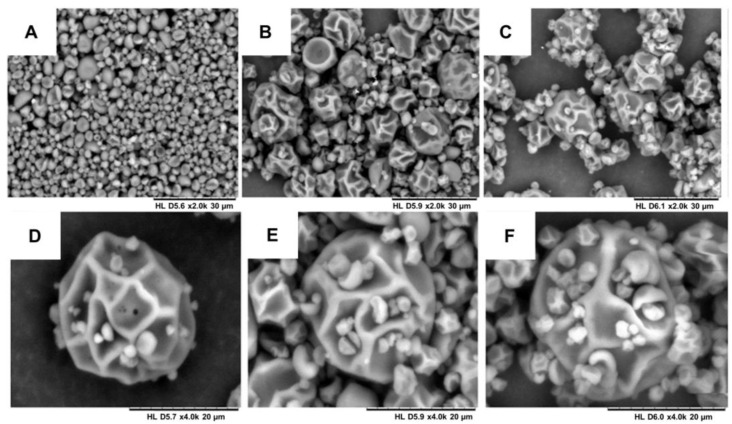
Scanning electron microscopy of the microparticles: (**A**) MP1—increase of 2000 times; (**B**) MP2—increase of 2000 times; (**C**) MP3—increase of 2000 times; (**D**) MP1—increase of 4000 times; (**E**) MP2—increase of 4000 times; and (**F**) MP3—increase of 4000 times.

Images of the solid microparticles MP1, MP2 and MP3 were obtained using scanning electron microscopy (SEM). Variations in the sizes of the microparticles were observed for both the placebo samples and those with incorporated bioactive compounds. The average sizes of the microparticles were as follows: 6.79 µm for MP1, 3.62 µm for MP2 and 5.57 µm for MP3. However, these differences in average size are not significantly expected to impact the performance of these systems in aqueous environments. Figure 4 illustrates the surface morphology of the produced particles. Most of the particles exhibited a rough surface, which can be attributed to the prolonged drying time and the high temperature (130 °C) required during the spray-drying process due to the substantial water content in the nanoemulsions and maltodextrin solutions. Previous studies that produced spray-dried lipid microparticles with smooth and homogeneous surfaces utilized lower temperature ranges, as they employed organic solvents, enabling drying at lower temperatures [47].

### 2.8. Differential Scanning Calorimetry and Thermogravimetry

**Figure 5:**
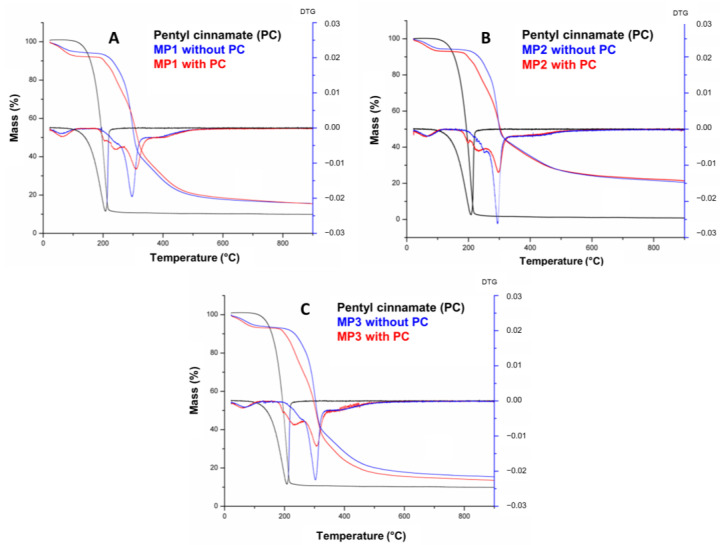
TG/DTG curves of pentyl cinnamate (PC) and solid microparticles MP1 (**A**), MP2, (**B**) and MP3 (**C**).

TG and DTG curves for the PC, together with the MP1, MP2 and MP3 with and without incorporated bioactive, are shown in Figure 5.

Figure 5 shows that the PC presents a single degradation step within a temperature range of 97–259.4 °C, totaling 97.8% mass loss. The temperature ranges of the mass loss stages, the percentage of mass involved at each stage and the total loss are detailed in Table 3. The curves of MP1, MP2 and MP3 exhibit a similar profile, indicating a correlation between their thermal events and those of maltodextrin, which is the predominant component in terms of mass in the microparticles. The first thermal event observed in all three MPs occurred within the temperature range of 22–118 °C and can be attributed to a first-order transition, such as evaporation, resulting in a water loss of approximately 5.8–6.9%. The onset of thermal degradation of the microparticles was observed at temperatures of 177–181 °C. A second, thermal event was observed in the temperature range of 177–330 °C, accompanied by a mass loss of approximately 47–56%. This mass loss can be attributed to the thermal decomposition of long molecular chains, polymerization processes and isomerization reactions associated with dehydration [48].

The decomposition temperature (Tonset) of PC increased from 97.0 °C to 177.0 °C, 180.0 °C and 181.0 °C in MP3, MP2 and MP1, respectively, demonstrating the thermal protection and stabilization effectiveness of the microparticles containing maltodextrin (Table S5, Supplementary Materials). These findings are consistent with the similar results previously reported for microparticles using this adjuvant [49,50]. The small difference of 4 °C between the Tonset values of the microparticles likely corresponds to the different substances present in the commercial lecithins and oils used in the nanoemulsion preparation and the interaction with PC and maltodextrin on microparticles obtained.

**Figure 6:**
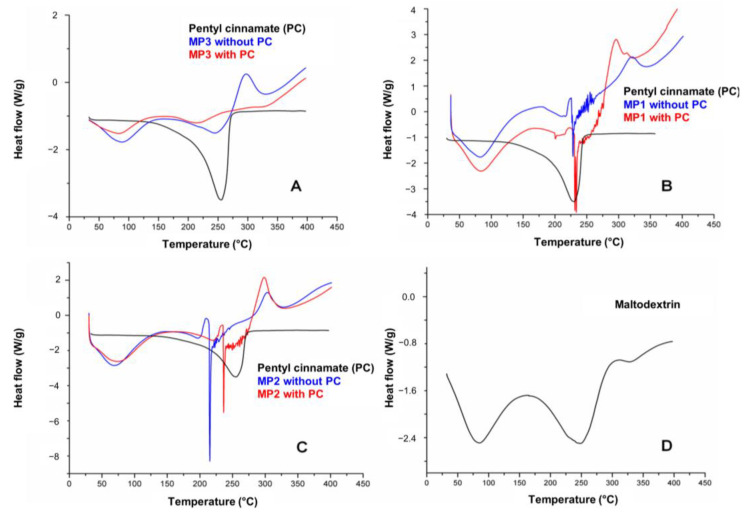
DSC curves of pentyl cinnamate (PC) with solid microparticles MP3 (**A**), MP1 (**B**), MP2 (**C**) and DSC curve of maltodextrin (**D**).

Figure 6 shows the DSC curves of PC, microparticles and maltodextrin, which were used as a drying aid in the atomization process. In the DSC curves for PC, a single endothermic event with a maximum peak at 254.74 °C was observed relating to the degradation of this compound. A table with DSC data of PC and solid microparticles MP1, MP2 and MP3 is in the Supplementary Materials (Table S6). The DSC curves of MP1, MP2 and MP3 showed similar behavior regarding the endothermic and exothermic events, which can be attributed to their main component, maltodextrin (Figure 6D). These events correspond to the observed mass loss in the TG curves, as the endothermic and exothermic events observed in MP1, MP2 and MP3 occur within the same temperature range as those observed in maltodextrin (82 °C, 245 °C). These events are associated with the loss of water, the initiation of thermal degradation of the MPs, and the thermal decomposition of the maltodextrin polymer chains [48].

## 3. Materials and Methods

### 3.1. Esterification of Cinnamic Acid

The PC was obtained by Fischer esterification of cinnamic acid (0.25 g) with *n*-pentanol (50 mL), using sulfuric acid (0.4 mL) as a catalyst. The reaction was carried out under reflux for 20 h and was monitored by thin-layer chromatography (Merck 60 F254, Darmstadt, Germany). Then, the *n*-pentanol was removed under reduced pressure and the product was added to a separatory funnel containing 20 mL of water. Extraction of the product was carried out with ethyl acetate (3 × 15 mL), and the combined organic phases were neutralized with 5% sodium bicarbonate. Afterward, it was washed with water and dried over anhydrous sodium sulfate. After evaporation in rotative evaporator, the PC was purified by silica gel 60-column chromatography (Merck 60, 230–400 mesh, Darmstadt, Germany) using hexane and ethyl acetate (7:3) as eluents. PC was obtained with 68.9% yield.

The purified ester was subjected to liquid chromatography coupled to mass spectrometry (Bruker, Model Elute UHPLC, Pump HPG1300) with a diode array detector (DAD) and a C18 column (100 mm × 2.1 mm, 1.8 μm, Bruker Intensity Solo 1.8). The developed method used acetonitrile (solvent B) and water (solvent A) as the mobile phase and was carried out in an isocratic manner with 73% of solvent B, in a total of 8 min at a flow rate of 0.30 mL/min, an oven temperature of 40 °C and an injection volume of 2 μL. The MS/MS detector used was a Q-TOF Bruker (Bremen, Germany), Compact model with electrospray ionization, 4.5 kV of capillarity, 200 °C dry temperature, 4 bar of nebulizer pressure, 9 L/min dry gas and 50–1000 mass range (*m*/*z*).

The chemical structure of PC was structurally characterized by infrared, ^1^H and ^13^C NMR spectroscopy. The hydrogen nuclear magnetic resonance (^1^H NMR) spectrum was obtained in a Bruker Ascend 300 spectrometer (Billerica, MA, USA). A probe with a frequency of 300 MHz (^1^H) was used. The sample was solubilized in 0.6 mL of deuterated chloroform. Chemical shifts (δ) were expressed in parts per million (ppm) and 65,536 points were used for spectrum acquisition and processing.

### 3.2. Nanoemulsion Preparation and Analysis

Oil-in-water nanoemulsions were prepared using an Ultra-Turrax homogenizer T-18 (IKA, Staufen, Germany) under a stirring speed at 10,000 rpm for 15 min. The temperature and proportion parameters between lecithins, Tween 80 (Vetec, Duque de Caxias, Brazil), sunflower oil (Mapric, São Paulo, Brazil) and water were changed to evaluate their influence on nanoemulsions.

Three different soy lecithins were used to prepare the nanoemulsions (NE1, NE2 and NE3): NE1 was prepared with soy lecithin containing 20% of phosphatidylcholine (LIPOID S20, Lipoid, Ludwigshafen, Germany), NE2 with soy lecithin containing 50% of phosphatidylcholine (PHOSAL SA 50+, Lipoid, Ludwigshafen, Germany), and NE3 with soy lecithin containing 70% of phosphatidylcholine (LIPOID S75-3, Lipoid, Ludwigshafen, Germany). The permission to access the Brazilian genetic patrimony was provided by SISGEN (AA84923). The proportion of lecithin was tested between 1.25 and 2.5% in NE1; between 3.5 and 5% in NE2; and between 1.0 and 1.5% in NE3. The temperatures tested were 25, 50 and 60 °C. PC was solubilized in sunflower oil to prepare the nanoemulsions and tween 80 was used as a co-surfactant between 0–1.75%. The composition data of each nanoemulsion can be found in Table S1 of the supplementary material.

In addition to PC, the NE1, NE2 and NE3 nanoemulsions without PC and with PC were analyzed by ^1^H NMR according to the methodology described in topic 3.1. The droplet size and distribution of the NE1, NE2 and NE3 nanoemulsions were obtained by dynamic light scattering (DLS) using a ZetaSize NanoZS instrument (Malvern Instruments, Malvern, UK). The samples were analyzed in triplicate and the dilution was in distilled water (1:100 (*v*/*v*)). Furthermore, the same instrument was used to evaluated the zeta potential at 25 °C by the samples’ electrophoretic mobility using a sodium chloride solution (0.1 mM) for samples’ dilution.

### 3.3. Larvicidal Activity of Pentyl Cinnamate and Nanoemulsions

Third-instar (L3) *Ae. aegypti* larvae (Rockefeller strain) aged (72–96 h after egg hatching) were collected from a mosquito colony maintained at the Laboratório de Farmacognosia Insectarium at the Universidade de Brasília without exposure to any insecticide. Larvicidal assays were conducted following the WHO guideline recommendations [51] with modifications [52]. For each bioassay, the temperature was maintained at 28 ± 2 °C and 70 ± 10% RH, with a 12 h light/dark photoperiod.

Quadruplicate assays, repeated three times using three different larvae lots from the same colony, were conducted in 12-well plates. Each well contained 10 L3 larvae, 3 mL of tap water and the respective PC compound diluted in DMSO (<2% dimethyl sulfoxide) or nanoemulsion (NE1, NE2 and NE3) diluted in tap water to achieve a final concentration of 50 µg/mL. Nanoemulsions without PC were also tested in parallel as the negative control. The organophosphate insecticide temephos (Sigma Aldrich, Buchs, Switzerland) was diluted in tap water (0.0016 to 0.0250 µg/mL) and tested in parallel as the positive control. After 24, 48 and 72 h of exposure, wells were inspected and the number of dead larvae used to determine the mortality percentage. Larvae with no movement, confirmed by light plate agitation, were considered dead. Samples causing ≥ 80% mortality were considered active.

Due to sample availability, the LC50 (µg/mL) values were determined in 12-well plates for PC (solubilized in DMSO) and the nanoemulsions: NE1, NE2 and NE3 (solubilized in dechlorinated tap water), using test concentrations ranging from 50 to 6.25 µg/mL. DMSO and the nanoemulsions without PC were also tested in parallel.

PC was evaluated at 50 µg/mL in a similar manner in cups, with each cup containing 25 L3 larvae in a final volume of 20 mL of tap water, following the same analytical procedure. The assay was repeated three times in quadruplicate using 3 larvae lots from the same colony. The PC compound LC50 (µg/mL) value was determined using concentrations ranging from 50 to 6.25 µg/mL (with <1% DMSO).

The residual activity of PC was determined in the laboratory by comparing the number of viable and dead larvae in the treated and control assays. Each assay consisted of 25 L3 larvae in each of four cups containing a final volume of 20 mL of dechlorinated tap water, together with the sample tested at 50 µg/mL. The PC compound was diluted in DMSO (<1% in tap water), which was also used as the negative control. An investigation of larvicidal activity was conducted by replacing the larvae every 24 h. The test was halted when no mortality was observed (no residual larvicidal activity) in accordance with the WHO guideline recommendations.

The average larvae mortality data were analyzed using the GraphPad Prism software (version 8.4.3) to calculate the LC50, LC90 and other 95% fiducial limits of upper and lower confidence. According to the WHO guidelines, tests with a control mortality > 5% must be corrected using Abbott’s formula and tests with a control mortality > 20% must be discarded.

### 3.4. Nanoemulsion Stability

A short-term stability study was carried out on PC nanoemulsions NE1, NE2 and NE3, using an adaptation of the previously described methodology [42]. The samples were stored at two different temperatures: 4 °C and 25 °C. This study was conducted for 30 days with the verification of visually observable instability phenomena, droplet size, polydispersity index (PDI), zeta potential and pH. In the visual analysis, we sought to observe the appearance of creaming, flocculation, or phase separation in nanoemulsions. The samples were analyzed in triplicate on days: 0, 1, 5, 15 and 30.

### 3.5. Spray Drying

Considering the particle size and PDI of the nanoemulsions produced, six systems were selected for the drying tests. The tests were carried out with two different drying aids: maltodextrin and *L*-leucine. The solution was prepared with a ratio of 1:4 *v*/*v* (nanoemulsion/adjuvant). Therefore, for the preparation of 100 mL of the solution to be subjected to spray drying, 75 mL of the solution with 20% (*w*/*v*) drying adjuvant were used when treated with maltodextrin, and 2% (*w*/*v*) when it was with *L*-leucine and 25 mL of the nanoemulsion. This mixture was subjected to magnetic stirring and dried in a spray-dryer atomizer (Yamato Scientific-model ADL311s, Nakatsu, Japan) using a 0.4 mm dosing nozzle, a blower ranging from 6.0–7.0, air atomization of 0.1 ppm, average flow of 2.0 mL/min and temperature controlled inlet (130–140 °C) and outlet (77–87 °C) for the evaluation of parameters such as yield and homogeneity of the collected powder. The solid microparticles called MP1 were produced from drying the NE1; MP2 and MP3 were produced from NE2 and NE3, respectively.

### 3.6. Fourier Transform Infrared (FTIR) Spectroscopy

Spray-dried solid microparticles (MP1, MP2 and MP3) containing PC at a concentration of 80.4 mg/L, together with the samples of the solid microparticles without PC and the PC, were subjected to Fourier transform infrared spectroscopic analysis (FTIR-ATR). The equipment used was an FTIR IR Prestige-21 spectrometer (Shimadzu, Tokyo, Japan) with attenuated total reflectance accessory employing a zinc selenide crystal. The spectra were obtained in duplicate, in a range of 700–4000 cm^−1^, with a resolution of 4 cm^−1^ and 20 scans.

### 3.7. Scanning Electron Microscopy

The morphology of the solid microparticles MP1, MP2 and MP3 was observed using a scanning electron microscope (SEM) (HITACHI—TM3000, Tokyo, Japan). The powder samples were fixed on carbon tape and inserted in a vacuum atmosphere, digitized and microphotographed. Microparticles’ mean sizes were determined by SEM using the Fiji/ImageJ with 32-bit Java software (version ij153) for size measurements.

### 3.8. Differential Scanning Calorimetry and Thermogravimetry

DSC curves of PC and solid microparticles MP1, MP2 and MP3 were recorded with a scanning calorimeter (Shimadzu—DSC-60, Japan) using a closed aluminum crucible with 2.0 mg of sample. Calibration was performed using an indium standard (156.6 ± 0.3 °C). In this experiment, the temperature increase was made in the range of 25–400 °C at a heating rate of 10 °C/min and a helium gas flow rate of 50 mL/min. DSC curves were evaluated using Shimadzu’s TASYS software TA-60WS version 1.40 to determine the melting temperature and enthalpy. The TG curves of the solid microparticles were obtained using a thermobalance (Shimadzu—DTG-60, Japan), with a nitrogen flow rate of 50 mL/min and a heating rate of 10 °C/min in the temperature range of 25–900 °C. The initial mass of the samples was 5.0 ± 0.5 mg, stored in an alumina crucible. The device was checked with the standard calcium oxalate monohydrate. TG curves were analyzed using Shimadzu’s TASYS software TA-60WS version 1.40.

## 4. Conclusions

Pentyl cinnamate was semi-synthesized and analyzed by spectroscopic and spectrometric techniques (^1^H NMR and HRMS) before biological activity evaluation. The com-pound showed activity against *Ae. aegypti* L3 larvae with LC50 19.9 µg/mL, which could be considered higher than other natural compounds, including methyl cinnamate. In addition, it was possible to prepare nanoemulsions using three different soy lecithins and the substances proposed in the aqueous and oily phases. The NE2 nanoemulsion showed increased stability over 30 days of observation. The nanoemulsions showed activity against *Ae. aegypti* L3 larvae with a mortality rate proportional to the exposure time. Higher temperatures were observed to be associated with PC degradation in solid microparticles. In summary, we demonstrated the use of PC and nanoemulsions as a potential larvicidal, and we also showed the compatibility of pentyl cinnamate with the solid microparticles produced and the increase in its thermal stability when incorporated into MPS.

## 5. Patents

BR1020200111990—Formulações líquidas e sólidas contendo cinamato de pentila, processo de obtenção e uso das mesmas para o controle de insetos hematófagos.

## Figures and Tables

**Table 1:** Average Droplet Size and PDI values of nanoemulsions in the stability test.

Sample	T (°C)		Time (Days)				
			Day 0 *	Day 1 *	Day 5 *	Day 15 *	Day 30 *
NE1	4	Size (nm) PDI	399 ± 66 0.91 ± 0.15	667 ± 132 0.79 ± 0.11	724 ± 403 0.81 ± 0.17	305 ± 46 0.69 ± 0.14	301 ± 37 0.74 ± 0.16
NE1	25	Size (nm) PDI	399 ± 66 0.91 ± 0.15	1481 ± 675 0.86 ± 0.11	1.404 ± 555 0.78 ± 0.08	610 ± 204 0.78 ± 0.10	651 ± 342 0.74 ± 0.09
NE2	4	Size (nm) PDI	158 ± 3 0.30 ± 0.03	160 ± 9 0.30 ± 0.05	169 ± 12 0.36 ± 0.08	200 ± 47 0.40 ± 0.10	207 ± 39 0.39 ± 0.11
NE2	25	Size (nm) PDI	158 ± 3 0.30 ± 0.03	158 ± 3 0.26 ± 0.02	233 ± 55 0.34 ± 0.09	191 ± 9 0.22 ± 0.06	267 ± 10 0.21 ± 0.04
NE3	4	Size (nm) PDI	460 ± 76 0.61 ± 0.06	460 ± 76 0.61 ± 0.06	456 ± 81 0.59 ± 0.09	578 ± 169 0.68 ± 0.10	594 ± 173 0.63 ± 0.12
NE3	25	Size (nm) PDI	460 ± 76 0.61 ± 0.06	556 ± 326 0.64 ± 0.16	485 ± 60 0.63 ± 0.13	1278 ± 407 0.91 ± 0.11	514 ± 332 0.67 ± 0.11

* The values are mean ± S.D (n = 3).

**Table 2:** Zeta potential (ZP) values of nanoemulsions in the stability test.

Sample	T (°C)		Time (Days)				
			Day 0 *	Day 1 *	Day 5 *	Day 15 *	Day 30 *
NE1	4	ZP (mV)	−65 ± 1	−60 ± 2	−56 ± 5	−56 ± 3	−59 ± 1
NE1	25		−65 ± 1	−59 ± 4	−59 ± 1	−58 ± 3	−57 ± 3
NE2	4	ZP (mV)	−28 ± 0	−29 ± 0	−34 ± 1	−29 ± 6	−32 ± 3
NE2	25		−28 ± 0	−38 ± 5	−36 ± 2	−35 ± 2	−36 ± 2
NE3	4	ZP (mV)	−57 ± 1	−51 ± 2	−46 ± 4	−40 ± 1	−44 ± 2
NE3	25		−57 ± 1	−45 ± 3	−47 ± 3	−42 ± 2	−47 ± 5

* The values are mean ± S.D (n = 3).

**Table 3:** TG data for pentyl cinnamate (PC) and solid microparticles produced with lecithins: MP1, MP2 and MP3.

Sample	1st Step	2nd Step	3rd Step	4th Step	5th Step	6th Step	Total
	Ti–Tf/°C (∆m%)	Ti–Tf/°C (∆m%)	Ti–Tf/°C (∆m%)	Ti–Tf/°C (∆m%)	Ti–Tf/°C (∆m%)	Ti–Tf/°C (∆m%)	(∆m%)
PC	97.00–259.39 (97.8)						97.80
MP1 without PC	31.94–132.21 (4.91)	167.46–286.13 (18.80)	286.13–320.99 (31.22)	320.99–791.38 (27.84)			82.77
MP1 with PC	21.82–109.72 (6.86)	180.59–220.95 (5.80)	220.95–282.46 (19.38)	282.46–330.62 (24.99)	330.62–476.60 (20.14)	476.60–893.71 (6.15)	83.32
MP2 without PC	24.44–113.01 (5.15)	180.56–270.93 (15.50)	270.93–309.40 (32.23)	309.40–474.47(17.74)	474.47–899.01 (7.96)		78.58
MP2 with PC	29.32–118.48 (5.89)	180.00–212.99 (4.64)	212.99–266.49 (16.88)	266.49–317.31 (25.57)	317.31–478.24 (16.91)	478.24–891.94 (6.70)	76.59
MP3 without PC	27.22–137.13 (5.53)	192.09–281.75 (17.17)	281.75–317.16 (33.18)	317.16–450.79 (19.65)	450.79–894.00 (7.53)		82.86
MP3 with PC	26.01–110.50 (5.84)	177.10–277.68 (27.50)	277.68–329.08 (28.83)	329.08–449.77 (16.52)	449.77–886.05 (6.32)		85.01

## Data Availability

Not applicable.

## Supplementary Materials

The supporting information can be downloaded at: https://www.mdpi.com/article/10.3390/ijms241512141/s1.
