# Supplementary material for: Nanoemulsions and Solid Microparticles Containing Pentyl Cinnamate to Control Aedes aegypti
Source: Int J Mol Sci. 2023 Jul 29;24(15):12141. doi: 10.3390/ijms241512141 (PMC10419096; doi:10.3390/ijms241512141)
Supplement: Supplementary file 1 [file ijms-24-12141-s001.zip › ijms-2434230-supplementary.pdf]

Nanoemulsions and solid microparticles containing pentyl cinnamate to control *Aedes aegypti*

Supplementary material

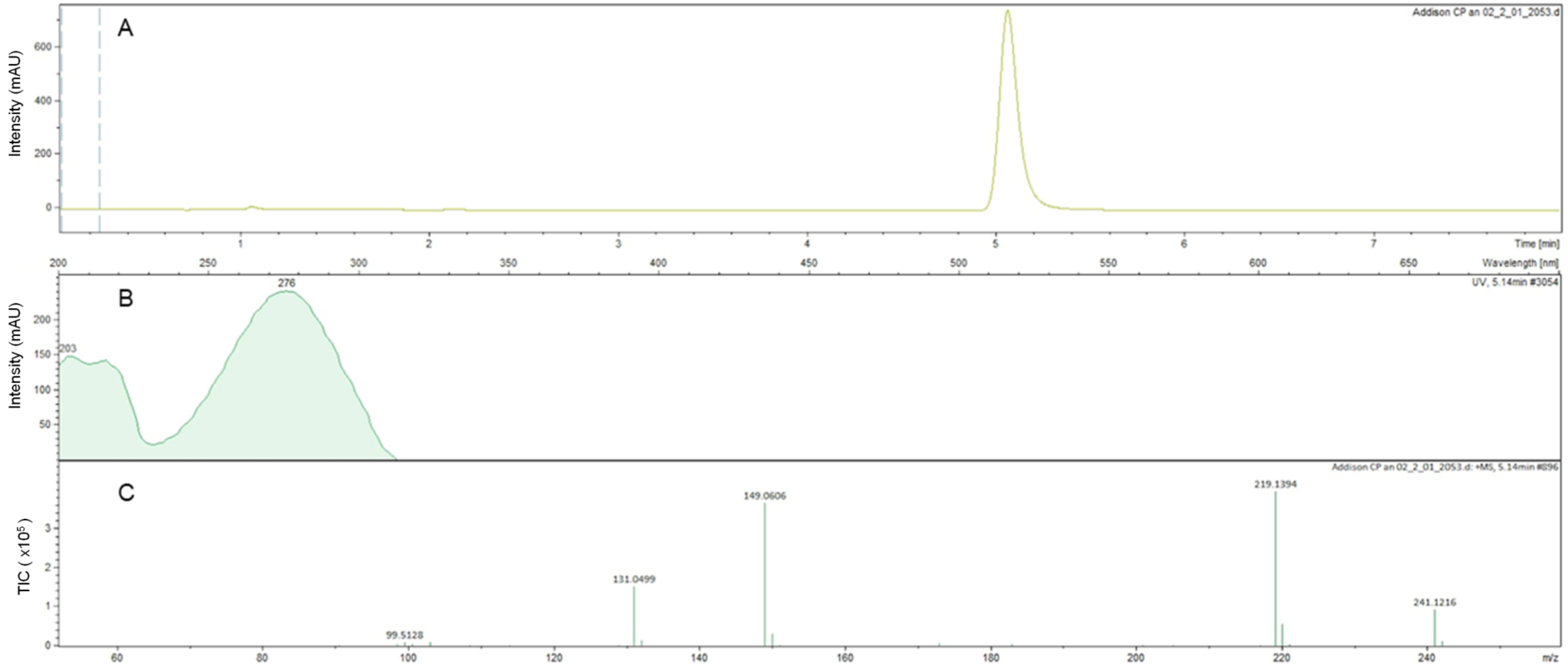

Figure S1. LC-MS/MS spectrum of pentyl cinnamate. In A: Total ion chromatogram obtained by the isocratic method in LC-MS/MS (ESI-QTOF), B and C: UV and mass spectrum spectrum for peak at 5.1 min, respectively. $[M+H]^+$ m/z 219.1394 (calcd. for $[C_{14}H_{19}O_2]^+$ m/z 219.1385, error 4.1 ppm).

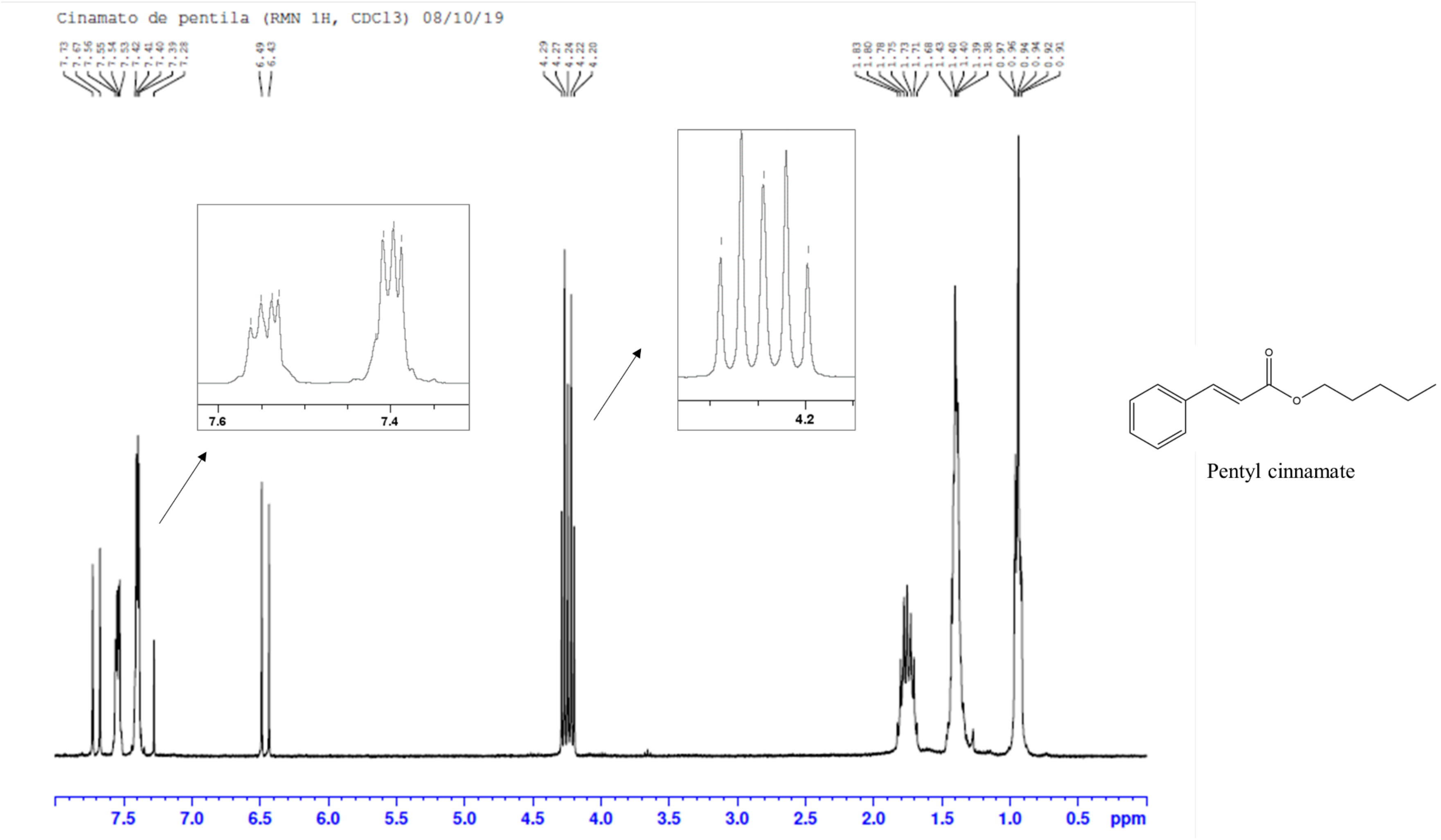

Figure S2. ^{1}H NMR (300 MHz) spectrum of pentyl cinnamate (PC) in $CDCl_3$.

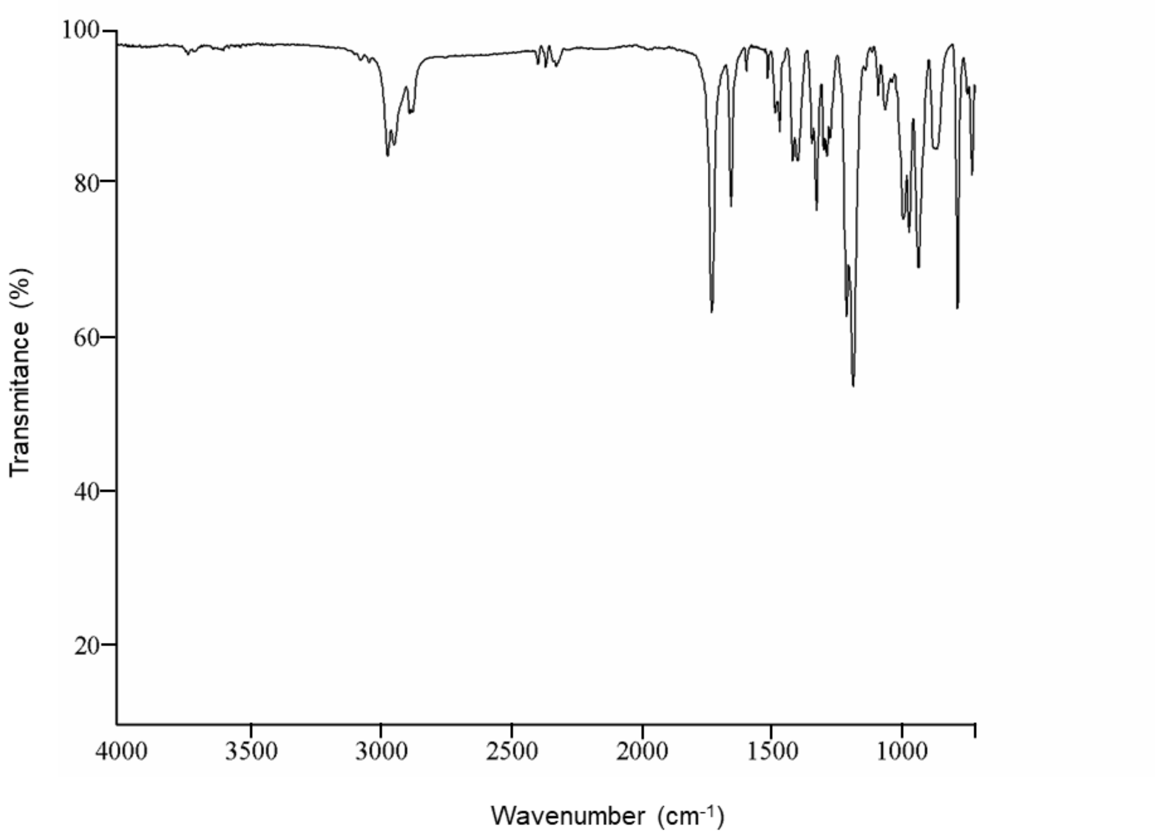

Figure S3. FTIR-ATR spectrum of pentyl cinnamate (PC) obtained in the range of 700-4000 cm^{-1}.

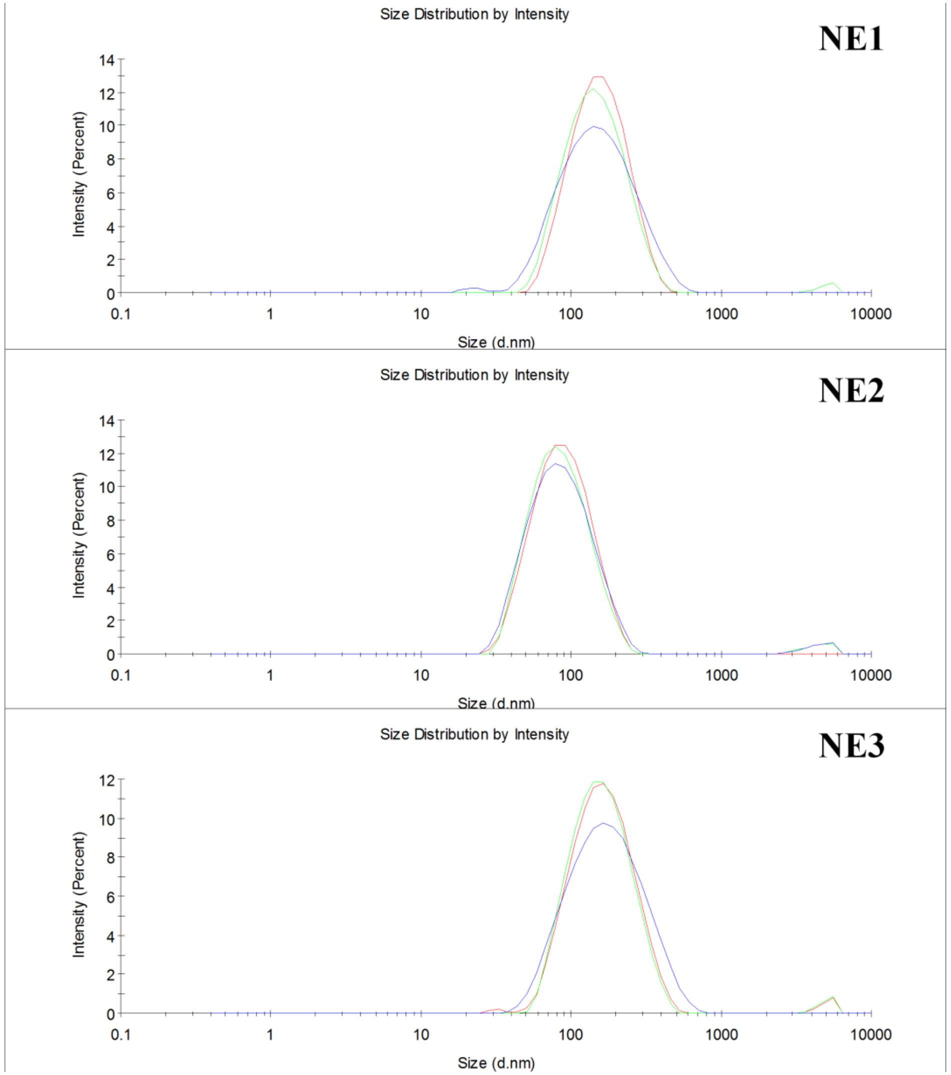

Figure S4. Size distribution of nanoemulsions NE1, NE2 and NE3 obtained by Dynamic light scattering (DLS).

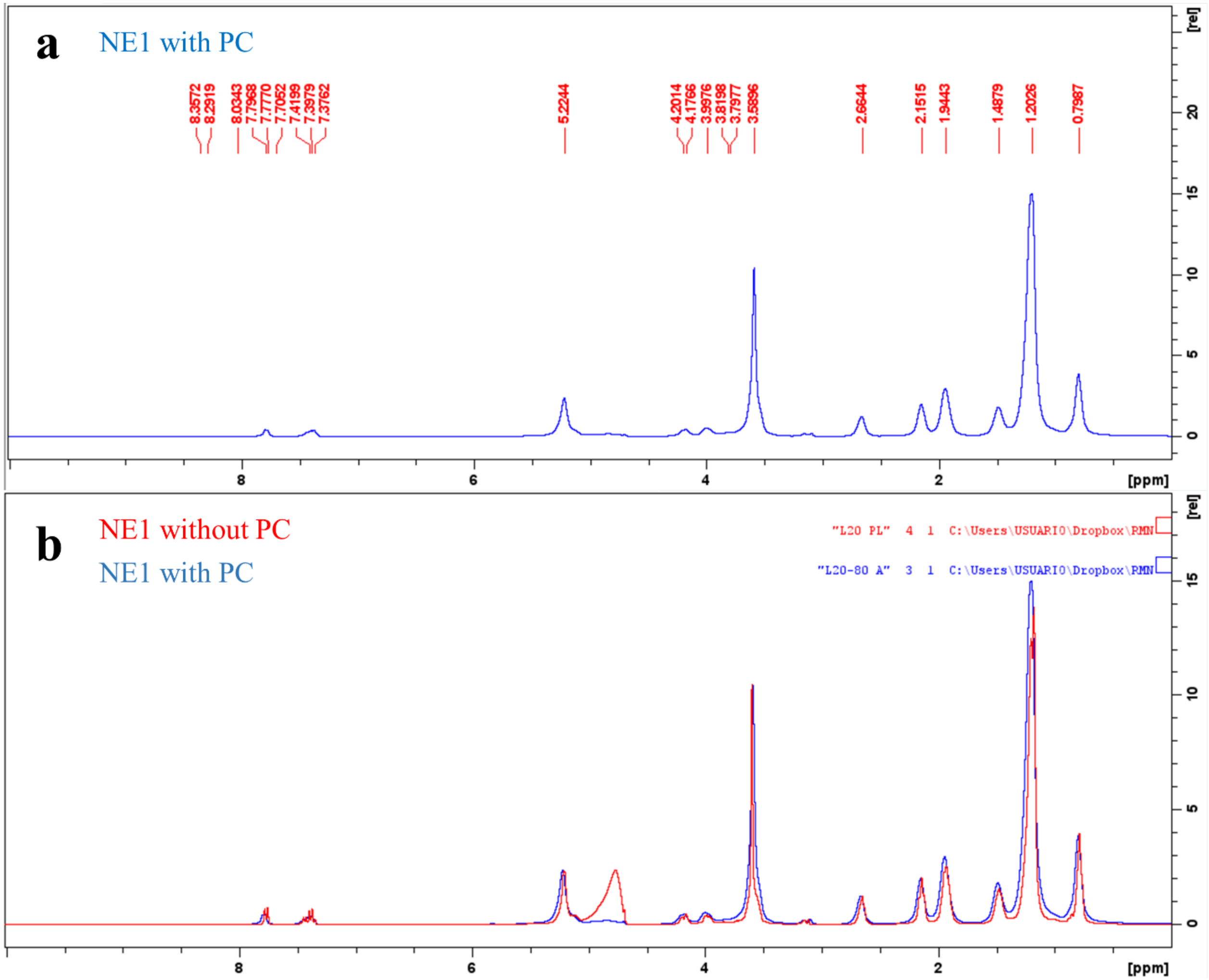

Figure S5. (a) ^{1}H NMR (300 MHz) spectra of NE1 with PC in D_2O and (b) superposition of ^{1}H NMR (300 MHz) spectra of NE1 with PC (blue) and without PC (red) in D_2O

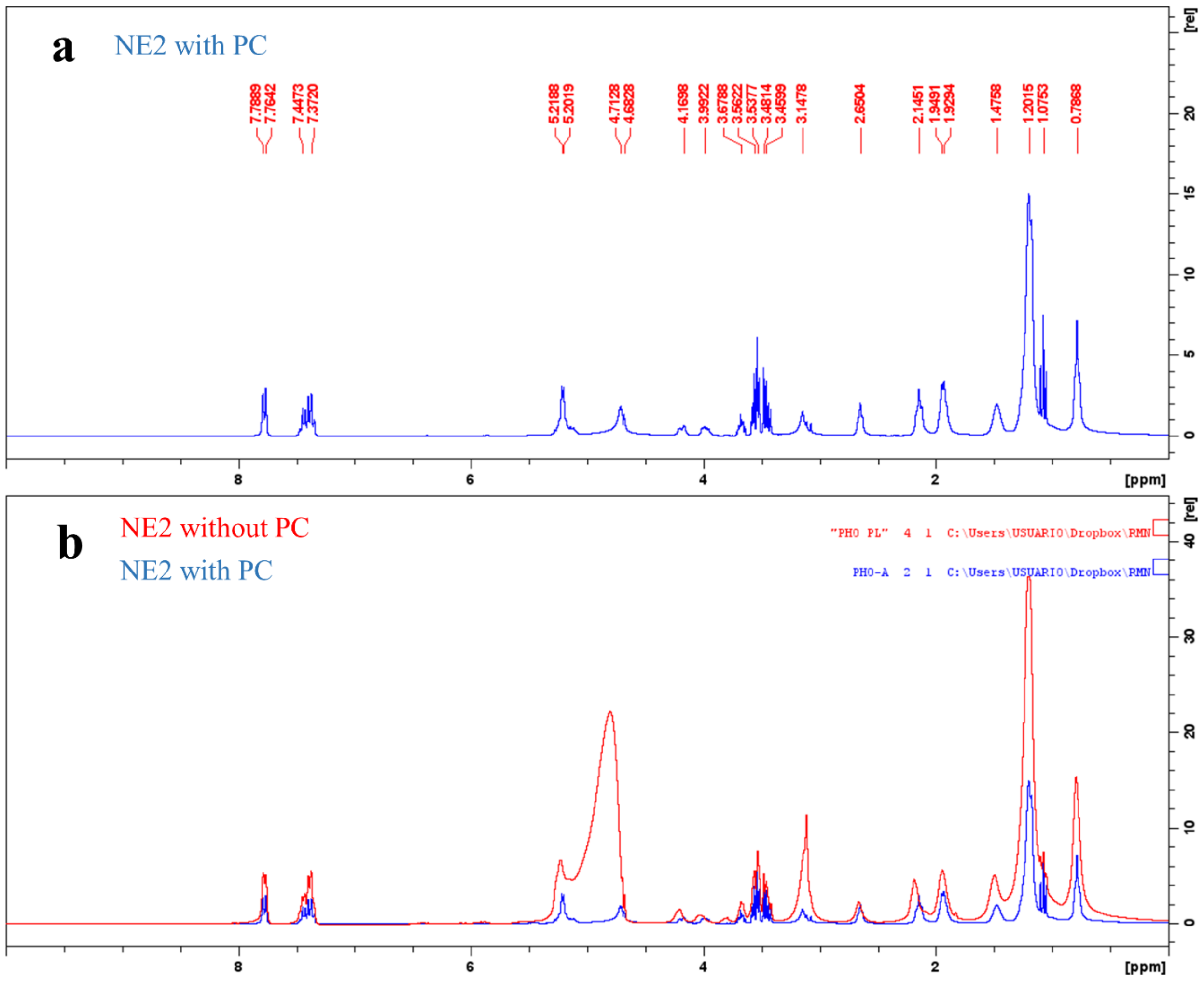

Figure S6. (a) ^{1}H NMR (300 MHz) spectra of NE2 with PC in D_2O and (b) superposition of ^{1}H NMR (300 MHz) spectra of NE2 with PC (blue) and without PC (red) in D_2O

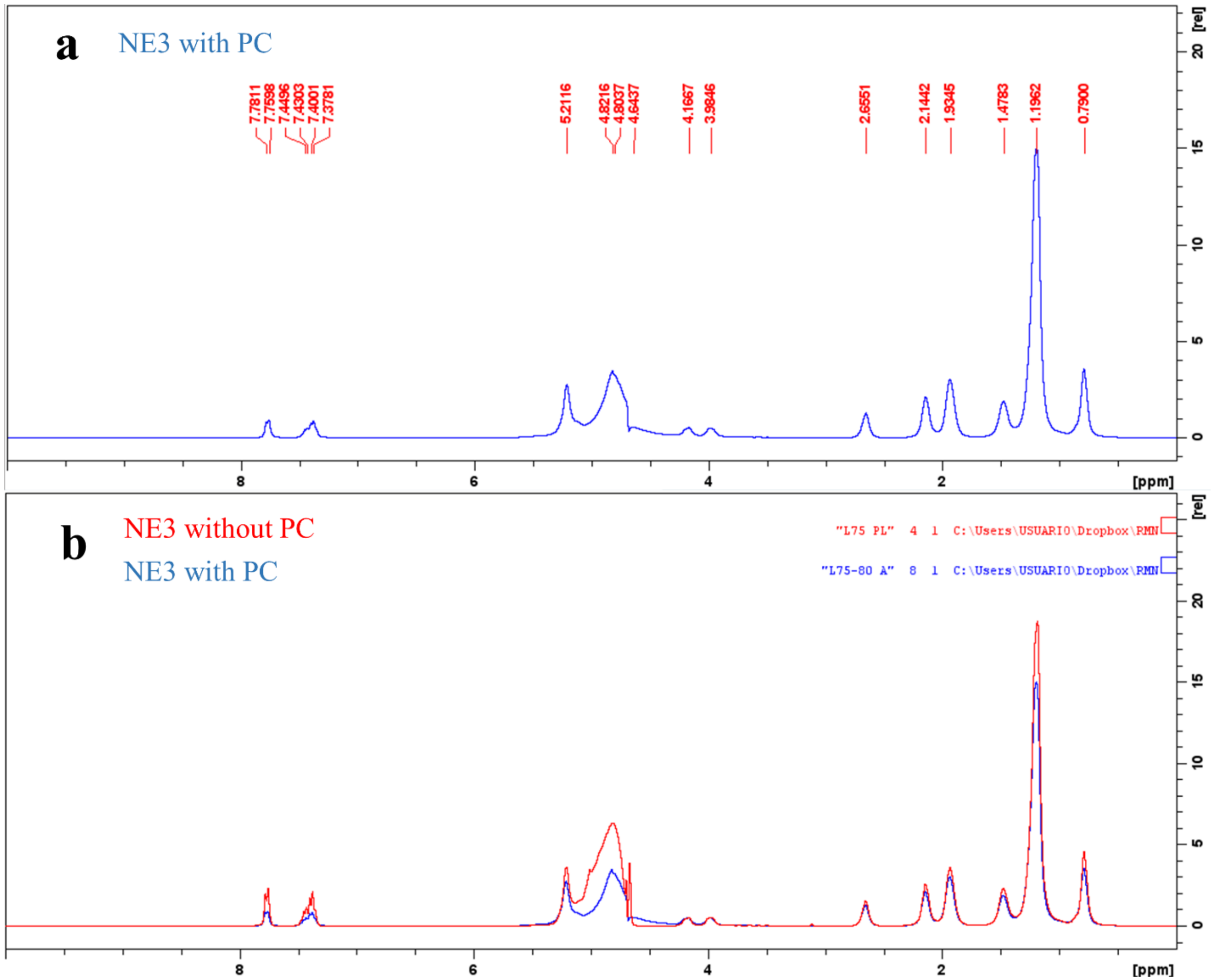

Figure S7. (a) ^{1}H NMR (300 MHz) spectra of NE3 with PC in D_2O and (b) superposition of ^{1}H NMR (300 MHz) spectra of NE3 with PC (blue) and without PC (red) in D_2O

Table S1 Composition of the nanoemulsions with the best results of particle size and PDI.

Sample	Sunflower oil (%)	Lecithin (%)	Tween 80 (%)	Water (%)	T. (ºC)
NE1 A	5.0	1.5	1.5	91.5	60.0
NE1 B	5.0	1.75	1.25	91.5	25.0
NE2 A	0.0	5.0	0.0	94.5	25.0
NE2 B	0.0	5.0	0.5	94.0	50.0
NE3 A	5.0	1.5	0.0	93.0	25.0
NE3 B	5.0	1.5	0.5	92.5	60.0

Table S2 NMR data assignments for PC ($CDCl_3$ 300/75 MHz)

Pentil cinnamate	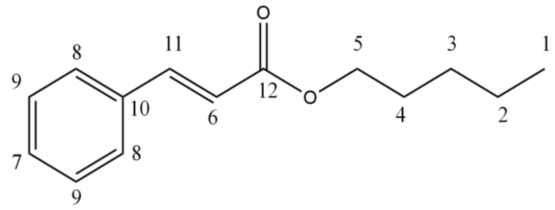	
**Carbon signal**	**δ ^{1}H**	**δ ^{13}C**
1	0.93 (3H, t, J=7.2 Hz)	14.1
2	1.38 - 1.43 (4H, m)	22.4
3		28.2
4	1.68 - 1.83 (2H, q, J=6.8 Hz)	28.5
5	4.20 - 4.29 (2H, m, J= 6.8 Hz)	64.8
6	6.46 (1H, d, J=16 Hz)	118.4
7	7.39 - 7.42 (3H, m)	128.1
8		129.0
9	7.53 - 7.55 (2H, dd)	130.3
10	-	134.5
11	7.70 (1H,d , J=16 Hz)	144.6
12	-	167.2

Table S3 Chemical shift (ppm) obtained by ^{1}H NMR (300 MHz) spectra of nanoemulsions NE1, NE2 and NE3 with PC and without PC.

Peak	NE1	NE1 with PC	NE2	NE2 with PC	NE3	NE3 with PC
1	7.781	7.787	7.786	7.788	7.779	7.781
2	7.755	7.762	7.763	7.764	7.756	7.759
3	7.473	-	7.449	7.447	7.450	7.449
4	7.449	7.449	7.428	-	7.428	7.430
5	7.426	7.427	7.397	-	7.399	7.400
6	7.398	7.397	7.374	7.372	7.376	7.378
7	7.373	7.374	5.227	5.218	5.207	5.211
8	7.350	7.349	-	5.201	5.010	-
9	5.216	5.213	4.800	-	4.816	4.821
10	5.206	-	-	4.712	4.806	4.803
11	4.771	4.766	-	4.682	4.697	-
12	-	4.695	4.203	-	4.667	4.643
13	4.196	4.196	-	4.169	4.194	-
14	4.165	4.163	4.026	3.992	4.165	4.166
15	4.004	4.001	3.674	3.678	3.986	3.984
16	3.987	3.984	3.661	-	2.653	2.655
17	3.968	3.965	3.560	3.562	2.142	2.144
18	3.951	-	3.533	3.537	1.931	1.934
19	3.598	3.599	3.478	3.481	1.476	1.478
20	-	3.149	3.457	3.459	1.184	-
21	-	3.112	-	3.147	-	1.196
22	2.651	2.649	3.112	-	0.788	0.790
23	2.142	2.139	2.664	2.650		
24	2.120	-	2.185	-		
25	1.948	1.928	-	2.145		

26	1.930	-	1.942	1.949		
27	1.478	1.474	-	1.929		
28	1.203	1.200	1.497	-		
29	1.177	1.175	-	1.475		
30	0.787	0.785	1.203	1.201		
31			1.073	1.075		
32			0.794	-		
33			-	0.786		

Table S4 Larvicidal activity of PC and nanoemulsions on larvae of *Aedes aegypti* after 24, 48 and 72 h.

Sample	Mortality (%)			Larvicidal Assay (LC_{50} µg/mL) (CI)		
	**24 h**	**48 h**	**72 h**	**24 h**	**48 h**	**72 h**
PC	100	-	-	22.0 (20.0 to 24.0)	20.2 (17.9 to 22.5)	19.9 (16.4 to 22.9)
NE1	37.3	74.7	88.0	> 50.0	24.7 (19.3 to 32.7)	19.4 (19.1 to 19.9)
NE2	54.7	74.7	89.3	> 50.0	<6.25	<6.25
NE3	72.0	96.0	98.6	33.8 (30.7 to 37.2)	16.3 (13.5 to 19.9)	11.9 (9.8 to 15.3)
DMSO	0	0	0	nd	nd	nd

nd – not determined; LC - Lethal concentration; CI - Confidence interval.

Table S5 Tonset of decomposition of PC and solid microparticles.

Sample	Tonset (ºC)
PC	97.0
MP1 PL	167.0
MP1 AT	181.0
MP2 PL	181.0
MP2 AT	180.0
MP3 PL	192.0
MP3 AT	177.0

* PL: solid microparticles without PC; AT: solid microparticles with PC.

Table S6 DSC data of PC and solid microparticles MP1, MP2 and MP3.

Sample	Peak (ºC)	Heat (J/g)	Peak (ºC)	Heat (J/g)	Peak (ºC)	Heat (J/g)
PC	254.70	-	-	-	-	-
MP1 PL	83.48	143.70	231.36	3.10	233.45	3.31
MP1 AT	70.19	181.77	214.57	0.81	216.12	2.63
MP2 PL	67.89	198.66	230.19	5.49	233.44	0.58
MP2 AT	69.00	156.13	215.17	14.92	-	-
MP3 PL	84.14	176.89	230.42	1.17	236.83	13.36
MP3 AT	83.09	-	214.12	-	-	-

* PL: solid microparticles without PC; AT: solid microparticles with PC.
